# Descending Colon Diverticulum Perforation Caused by Migration of a Pancreatic Spontaneous Dislodgement Stent for Prophylaxis of Post-endoscopic Retrograde Cholangiopancreatography (ERCP) Pancreatitis

**DOI:** 10.7759/cureus.98923

**Published:** 2025-12-10

**Authors:** Yutaro Naka, Takashi Goto, Shimpei Uchida, Takanobu Sugase, Rintaro Koga

**Affiliations:** 1 Surgery, Koga General Hospital, Miyazaki, JPN; 2 Internal Medicine, Koga General Hospital, Miyazaki, JPN

**Keywords:** colon diverticulum, colon perforation, endoscopic retrograde cholangiopancreatography, pancreatic spontaneous dislodgement stent, post-ercp pancreatitis (pep), stent migration

## Abstract

Pancreatitis following endoscopic retrograde cholangiopancreatography (ERCP), known as post-ERCP pancreatitis (PEP), is the most common and potentially serious complication of the procedure. Pancreatic spontaneous dislodgement stents (PSDSs) are widely used to reduce the incidence of PEP in high-risk patients. Although these stents are generally safe, migration-related complications such as intestinal perforation are extremely rare but potentially life-threatening.

We present a case of descending colon perforation caused by migration of a PSDS placed for PEP prophylaxis. An 87-year-old man underwent ERCP for common bile duct stones, during which a 5 Fr, 3 cm straight-type PSDS was placed to prevent pancreatitis. Nineteen days later, he developed a fever, and computed tomography revealed perforation of the descending colon caused by the migrated stent. Laparoscopic exploration followed by open surgery confirmed the perforation and peritonitis. Partial colectomy with functional end-to-end anastomosis was performed, and the patient recovered without complications.

This case highlights a rare but critical complication of PSDS use, particularly in patients with colonic diverticula or other anatomical variations. It emphasizes the importance of recognizing the risks associated with PSDS placement, conducting appropriate follow-up, ensuring early detection of complications, and facilitating timely intervention. Additionally, development and selection of safer stent designs may help reduce the risk of migration-related adverse events in the future.

## Introduction

Post-endoscopic retrograde cholangiopancreatography (ERCP) pancreatitis (PEP) is the most common and potentially serious complication of ERCP. The prophylactic placement of a pancreatic spontaneous dislodgement stent (PSDS) is widely supported as an effective and safe strategy to reduce the incidence of PEP, particularly in high-risk patients, and its use has become increasingly common in recent years [1,2]. This strategy is also endorsed by Japanese and European guidelines [3,4].

PSDS devices are designed to spontaneously dislodge within a few days and transit through the gastrointestinal tract to be excreted in the stool. Because these stents migrate through the bowel, pre-existing anatomical abnormalities may influence the risk of complications during their passage. Colon diverticula, which are common in elderly individuals, are known sites of gastrointestinal perforation and are reported to perforate more frequently than other colonic segments.

However, serious complications such as intestinal perforation due to migration of these stents are rarely reported but can be life-threatening. We believe this to be the first reported case of colon diverticulum perforation caused by PSDS impaction.

Herein, we describe the clinical course and management of an 87-year-old man who developed descending colon perforation following PSDS placement for PEP.

## Case presentation

An 87-year-old man was transported to the hospital due to fever, chills, and right upper quadrant pain. He had a medical history of chronic heart failure, atrial fibrillation, chronic obstructive pulmonary disease, and aortic valve replacement. Despite these conditions, he remained independent in activities of daily living and showed no cognitive decline.

**Figure 1:**
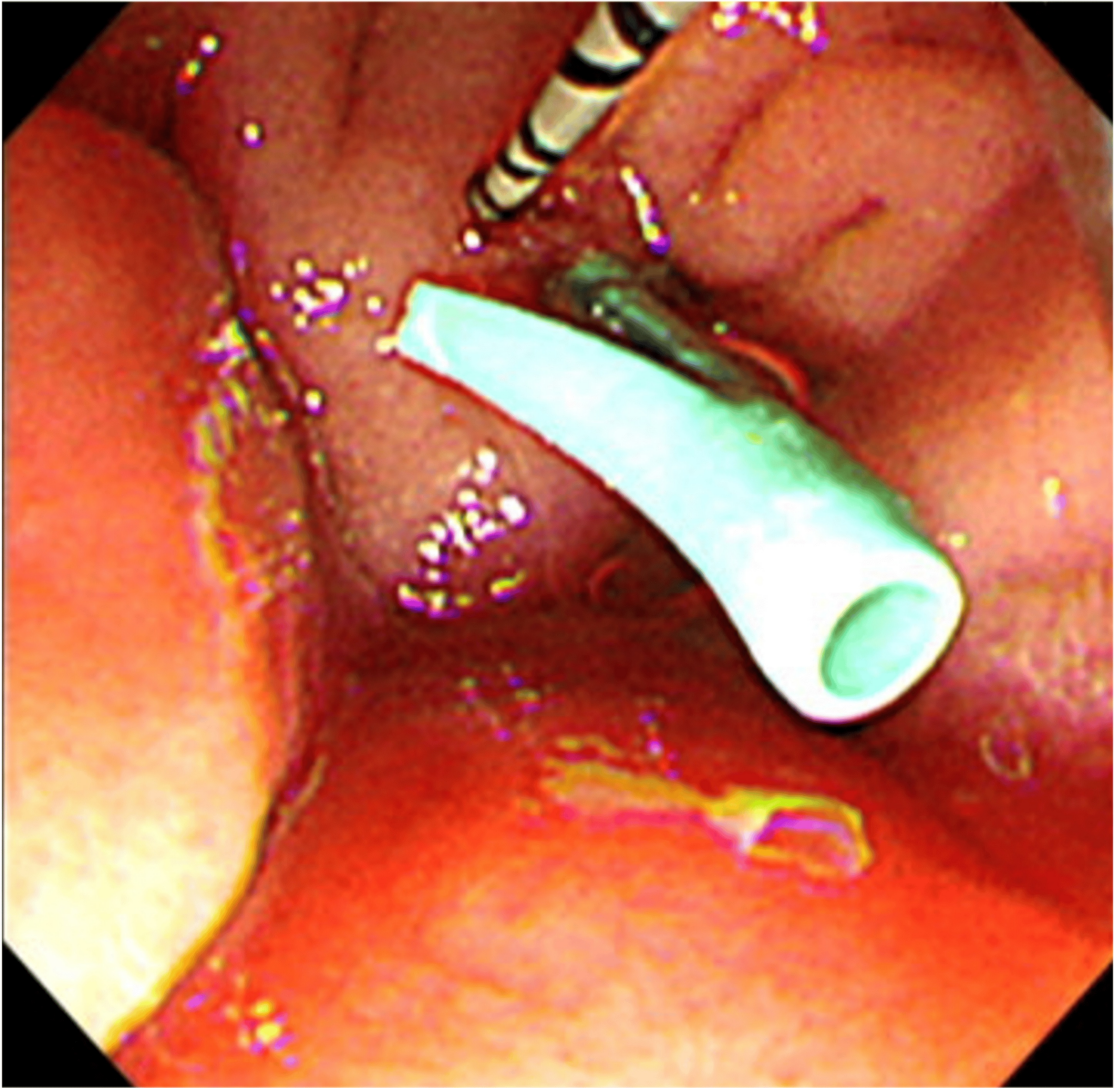
A PSDS was placed during emergency ERCP Emergency ERCP was performed on the day he was transported, during which an ENBD tube (5 Fr) and a PSDS was placed. The PSDS used was a polyethylene 5F diameter, 3 cm in length, straight-type stent, unflanged on the pancreatic ductal side with 2 flanges on the duodenal side (GPDS-5-3; Cook Japan, Tokyo, Japan). ERCP, emergency endoscopic retrograde cholangiopancreatography; ENBD, endoscopic nasobiliary drainage; PSDS, pancreatic spontaneous dislodgement stent

Computed tomography (CT) revealed common bile duct (CBD) stones with associated ductal dilation. Emergency ERCP was performed on the same day, during which an endoscopic nasobiliary drainage tube (5 Fr) and a PSDS were placed (Figure 1). The PSDS used was a polyethylene 5 Fr, 3 cm straight-type stent, unflanged on the pancreatic ductal side with two flanges on the duodenal side (GPDS-5-3; Cook Japan, Tokyo, Japan). Rectal nonsteroidal anti-inflammatory drugs NSAIDs were not used for PEP prophylaxis in this case.

**Figure 2:**
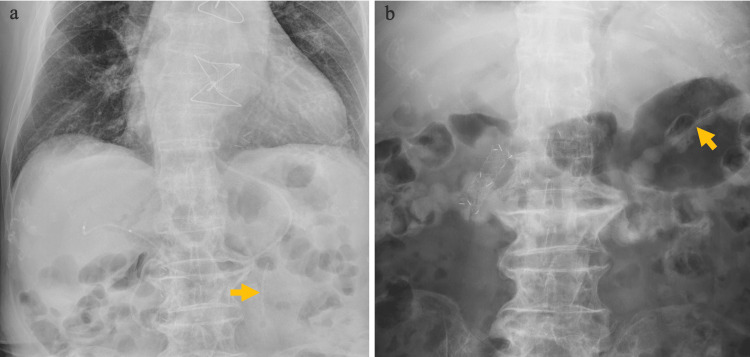
Abdominal X-ray images showing uncomplicated progression of the PSDS X-ray images taken on the 10th (a) and 12th (b) days confirmed an uncomplicated progression of the PSDS (yellow arrows) through the intestinal tract. PSDS, pancreatic spontaneous dislodgement stent

On day 4, a second ERCP and endoscopic sphincterotomy (EST) were performed, successfully extracting the CBD stones. The pancreatic stent had naturally dislodged during the procedure. An ERBD tube (7 Fr, 7 cm, pigtail) was placed. On day 7, another emergency ERCP was required due to post-EST bleeding. A fully covered self-expandable metal stent (10 mm, 4 cm) was placed in the bile duct. On day 10, the patient resumed meals. X-ray imaging on the 10th and 12th days confirmed an uncomplicated progression of the PSDS through the intestinal tract (Figure 2).

On the 17th day, the patient reported only mild abdominal discomfort without fever or significant abdominal pain. Laboratory tests showed a white blood cell count (WBC) of 5600/μL with 61% neutrophils and a C-reactive protein (CRP) level of 0.53 mg/dL. Because these symptoms and laboratory findings did not suggest an acute inflammatory process, additional imaging was not performed.

**Figure 3:**
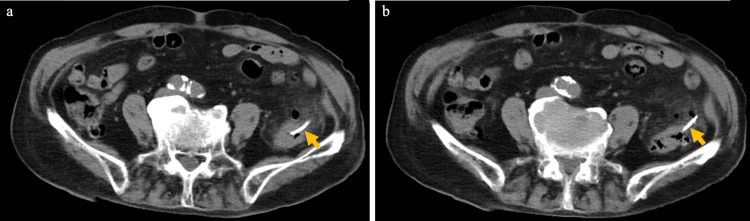
A perforation of the descending colon caused by the migrated PSDS On the 19th day, CT revealed perforation of the descending colon caused by the migrated PSDS (yellow arrows). CT, computed tomography; PSDS, pancreatic spontaneous dislodgement stent

However, on the 19th day, the patient developed a fever. Laboratory findings revealed a WBC count of 8100/μL with 72.3% neutrophils and a CRP level of 7.77 mg/dL. CT demonstrated perforation of the descending colon caused by the migrated pancreatic stent (Figure 3). He was diagnosed with generalized peritonitis secondary to colonic perforation, classified as Hinchey stage IV, necessitating emergency surgical intervention.

**Figure 4:**
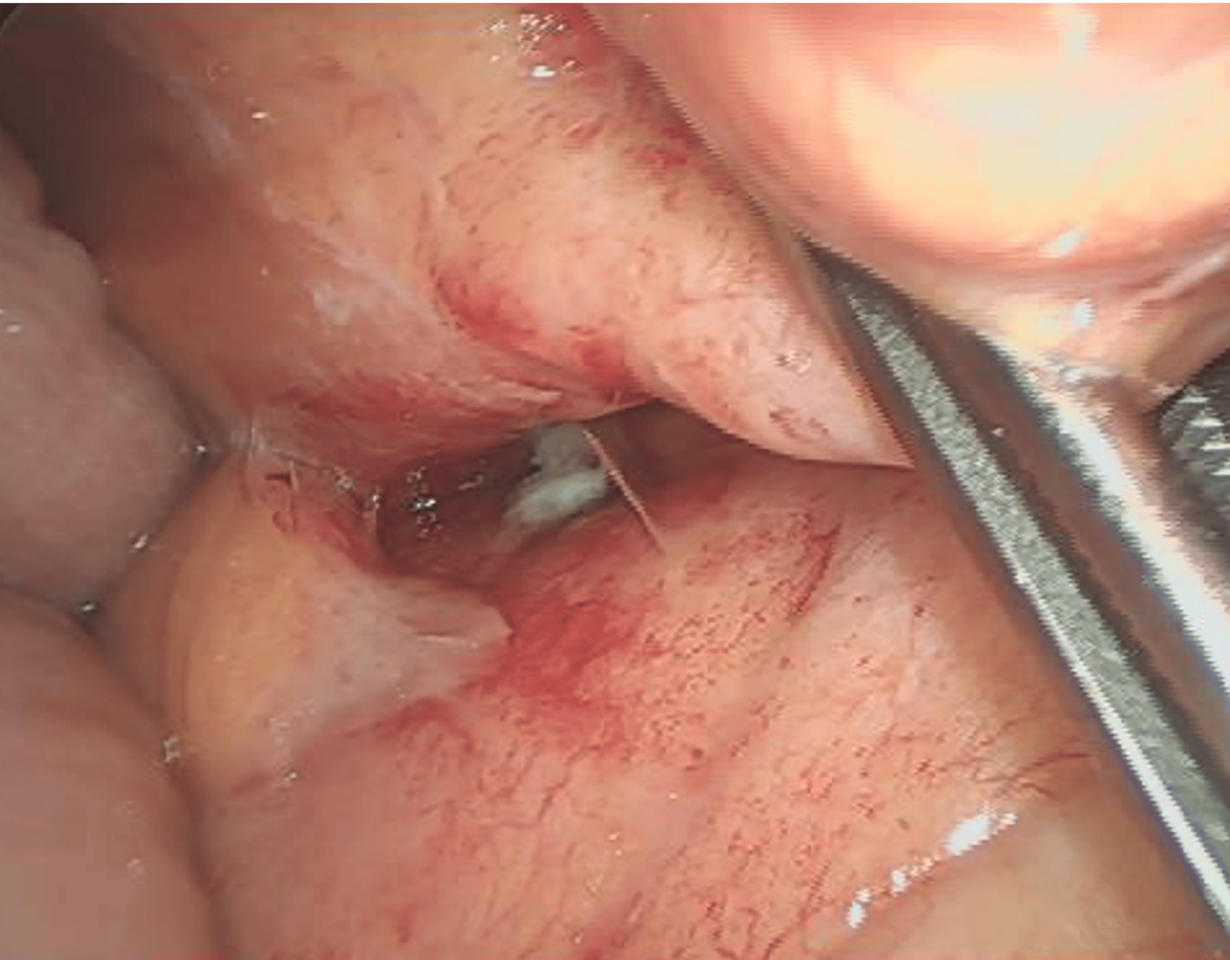
Intraoperative findings Perforation of the stent and inflammatory adhesions around the stent were identified through laparoscopic exploration.

We began with laparoscopic exploration of the abdominal cavity, identifying the stent-related perforation, adhesions, and turbid ascites from peritonitis, and then transitioned to open surgery (Figure 4). The perforation site was identified, and the descending colon was mobilized, followed by partial colectomy. The bowel was reconstructed using a functional end-to-end anastomosis, and the abdominal cavity was thoroughly irrigated before closure.

**Figure 5:**
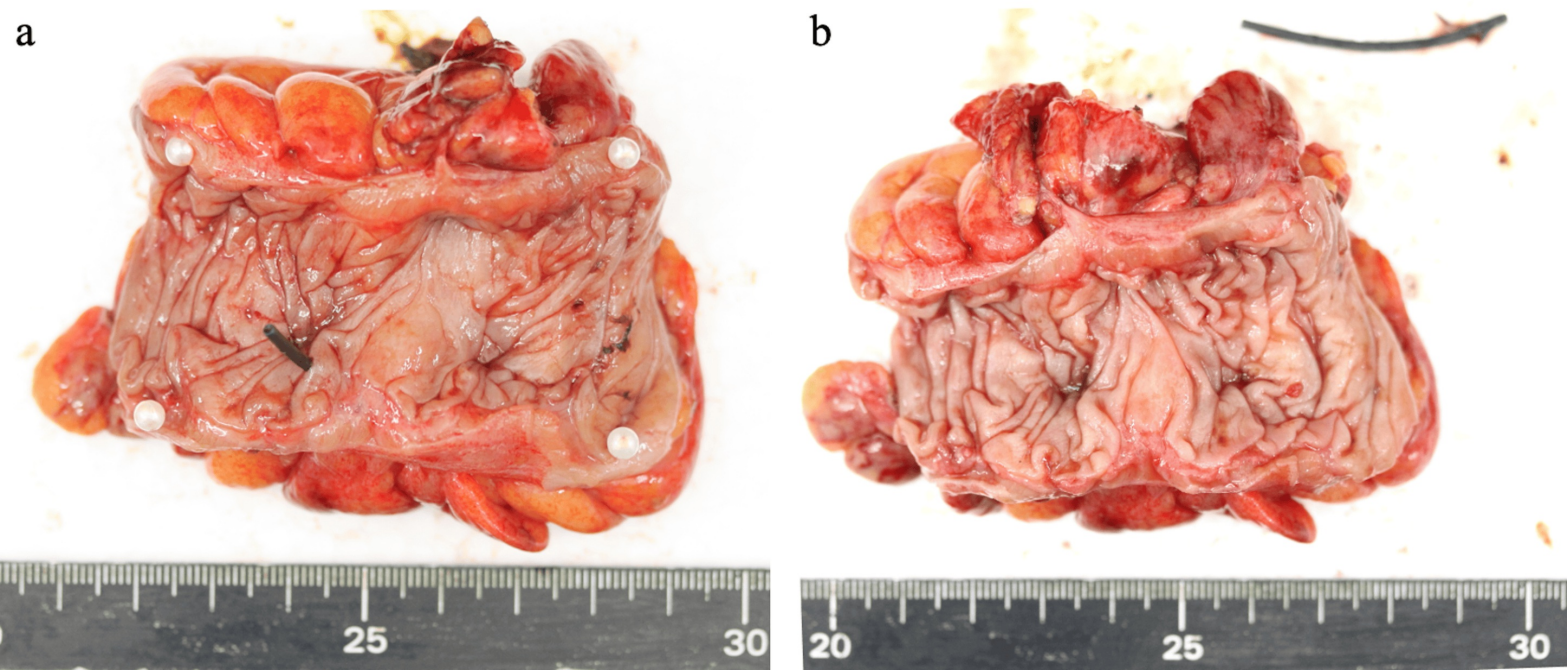
Resected specimen The perforation was caused by the flanged end of the spontaneously dislodged pancreatic duct stent embedding into the diverticulum. (a) The stent was found to be impacted within a diverticulum. (b) Image after stent removal.

The operative time was 192 minutes with minimal blood loss. Examination of the specimen revealed that the perforation occurred at a diverticulum, where the stent had become lodged (Figure 5). The flanged end of the stent was impacted within the diverticulum.

**Figure 6:**
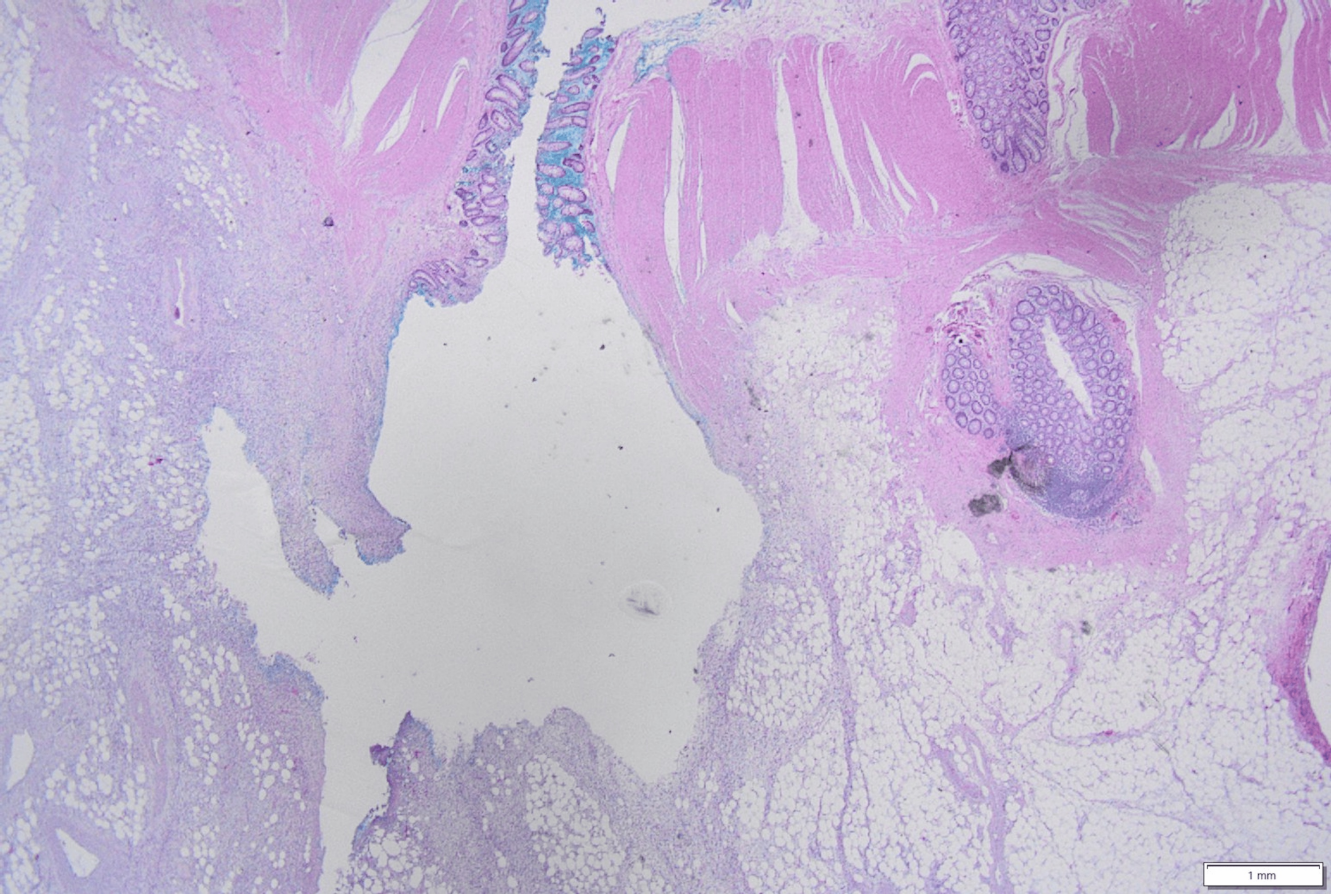
Histopathological findings of the perforated colonic diverticulum Low-power photomicrograph showing several diverticula in the colonic wall with mixed infiltration of neutrophils and lymphocytes around a diverticulum. Patchy neutrophilic infiltration is present in the subserosal layer, and fibrinomucinous material containing neutrophils is partly noted on the serosal surface.

Histopathological examination confirmed diverticular perforation caused by the migrated stent, consistent with the macroscopic findings. Mixed infiltration of neutrophils and lymphocytes was observed around the diverticulum, with patchy neutrophilic infiltration in the subserosal layer and fibrinomucinous material containing neutrophils partly on the serosal surface (Figure 6). No evidence of malignancy was found.

Oral intake of fluids and medications was resumed on postoperative day 2, and solid food intake on day 5. The patient was discharged on day 22 without complications.

## Discussion

PSDSs have been widely recognized as an effective strategy to reduce the incidence of PEP, particularly in high-risk patients, as demonstrated by multiple studies and supported by clinical guidelines [1-4]. In most cases, PSDSs have been reported to dislodge from the pancreatic duct and are subsequently expelled from the body with stool.

A study involving 203 cases using a 5 Fr, 3 cm straight PSDS, which was unflanged on the pancreatic ductal side but had two flanges on the duodenal side (GPDS-5-3; Cook Endoscopy, Inc, Winston-Salem, NC), reported a stent placement success rate of 91.6% and a spontaneous dislodgement rate of 95.7% within three days, with a mean dislodgement time of 1.8 days (range: 0-4 days) [1].

If a pancreatic stent fails to dislodge, mechanical irritation may cause pancreatic duct changes or pancreatitis, warranting endoscopic removal [5].

According to previous studies, overall prophylactic pancreatic stent-related complications have been reported in 4.4% of patients. The reported adverse events include any infection (3.0%), bleeding (2.5%), cholangitis and cholecystitis (3.1%), necrosis (0.4%), pancreatic duct perforation (0.8%), stent migration (4.9%), stent occlusion (7.9%), pancreatic pseudocyst formation (3.0%), and retroperitoneal perforation (1.2%) [2]. These data include various types of stents, such as PSDSs and other non-PSDS stents.

Intestinal perforation caused by PSDS is exceedingly rare. To date, only three cases involving the duodenum or jejunum have been documented: (i) a jejunal perforation caused by adhesions between jejunal loops secondary to peritoneal carcinomatosis, (ii) a perforation due to impaction in a periampullary diverticulum, and (iii) a jejunal perforation associated with peritoneal dissemination of colorectal cancer [6-8].

To facilitate comparison, these previously reported cases can be summarized as follows: all involved either malignant conditions or anatomical alterations around the small intestine, and none occurred in a benign colonic diverticulum. This highlights the unique nature of the present case.

In this case, the PSDS (5 Fr, 3cm, single-flanged) migrated and became lodged in a colonic diverticulum, resulting in perforation. The perforation was caused by the flanged end of a PSDS embedding into a diverticulum. Its flange may become caught within the diverticular lumen, making spontaneous dislodgement difficult.

Diverticula, which are common in elderly individuals, constitute a benign but significant anatomical risk factor for impaction because they can trap the flanged end of a stent and prevent spontaneous dislodgement. This highlights the importance of evaluating patient-specific anatomical risks before ERCP.

Smooth progression of the stent typically poses no issue. However, if a stent becomes lodged, fails to advance, or if symptoms such as abdominal pain or fever arise, early evaluation is warranted. Lower gastrointestinal endoscopy is a minimally invasive and effective method for retrieving a migrated PSDS.

**Figure 7:**
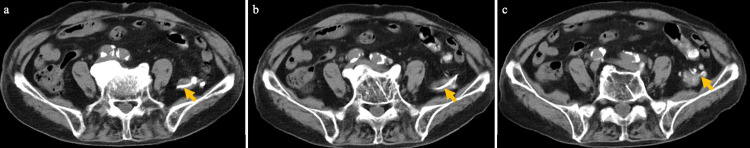
CT suggested that the stent might have been lodged in a diverticulum Retrospectively, the CT on day 14 in this case did not show perforation but suggested that the stent might have been lodged in a diverticulum. CT, computed tomography

Retrospectively, the follow-up CT obtained 14 days after the initial ERCP did not reveal a perforation but suggested that the stent might have been lodged in a diverticulum (Figure 7).

Although the patient noted mild abdominal discomfort on day 17, the nonspecific symptoms and normal inflammatory markers contributed to the diagnostic delay. As a result, additional imaging was not pursued, and the progression toward perforation remained unrecognized until it became clinically apparent. For patients with anatomical risk factors such as diverticula, even subtle abdominal discomfort may warrant earlier imaging, as timely detection of stent impaction could prevent progression to perforation.

While routine imaging follow-up for all patients with PSDSs may not be feasible in daily practice due to limited resources, selective surveillance of high-risk individuals, such as those with diverticula, adhesions, or peritoneal dissemination, is essential. Since most PSDSs spontaneously dislodge within a few days (with a reported rate of 95.7% within three days [1]), targeted follow-up using abdominal X-ray or CT starting a few days after insertion in these high-risk patients can confirm appropriate stent progression and detect early signs of migration or impending complications.

PSDSs are available in various sizes and configurations, including straight, pigtail, and flanged designs. Each type has specific advantages and limitations depending on the patient's anatomical characteristics and clinical context. However, there is currently no established consensus regarding the optimal stent design. With regard to diameter and length, current evidence supports the use of a 5 Fr, 3 cm stent [9,10].

In our case, the single-flanged end became lodged in a diverticulum, which impeded spontaneous dislodgement and caused the stent to embed progressively deeper within the diverticular lumen, ultimately resulting in perforation. An unflanged stent would likely have slipped out of the diverticulum spontaneously after transient impaction. Therefore, for patients with known colonic diverticula, selecting a non-flanged design may help reduce the risk of impaction-related perforation.

Future developments, such as fully biodegradable stents or devices with enhanced anchoring characteristics, may help reduce migration-related complications. However, further prospective evaluation is needed.

Taken together, these considerations suggest that preventing PSDS-related perforation requires three key measures: selective imaging follow-up in high-risk patients, choosing stent designs based on individual anatomy, and continued development of safer stent technologies.

## Conclusions

In conclusion, we encountered a rare case of colonic perforation caused by the migration of a PSDS placed for PEP prophylaxis. This case highlights the importance of recognizing the potential risks associated with PSDSs, particularly in patients with diverticula, adhesions, or peritoneal dissemination.

Clinicians should pay special attention to these high-risk patients and consider early or selective imaging follow-up even when symptoms are mild to facilitate prompt detection of stent-related complications. When appropriate, timely interventions such as lower gastrointestinal endoscopy can help prevent severe outcomes, including gastrointestinal perforation. In this case, although non-surgical management would have been preferable, surgical intervention ultimately became necessary, and early surgery enabled a favorable recovery, underscoring how vigilant monitoring and timely intervention can improve patient safety and clinical outcomes.
